# Supplementary material for: Pyruvate Dehydrogenase Kinase 1 inhibition mediated oxidative phosphorylation enhancement in cartilage promotes osteoarthritis progression
Source: BMC Musculoskelet Disord. 2023 Jul 20;24:597. doi: 10.1186/s12891-023-06585-6 (PMC10357736; doi:10.1186/s12891-023-06585-6)
Supplement: Supplementary file 1 — Additional file 1. [file 12891_2023_6585_MOESM1_ESM.zip › MF.Gsea.1653627316242/GOMF_OXIDOREDUCTASE_ACTIVITY_ACTING_ON_THE_ALDEHYDE_OR_OXO_GROUP_OF_DONORS.html]

Details for gene set GOMF\_OXIDOREDUCTASE\_ACTIVITY\_ACTING\_ON\_THE\_ALDEHYDE\_OR\_OXO\_GROUP\_OF\_DONORS[GSEA]

|  || Dataset | 55457\_collapsed\_to\_symbols.cls.cls #OA\_versus\_normal.cls.cls #OA\_versus\_normal\_repos |
| Phenotype | cls.cls#OA\_versus\_normal\_repos |
| Upregulated in class | OA |
| GeneSet | GOMF\_OXIDOREDUCTASE\_ACTIVITY\_ACTING\_ON\_THE\_ALDEHYDE\_OR\_OXO\_GROUP\_OF\_DONORS |
| Enrichment Score (ES) | 0.39468312 |
| Normalized Enrichment Score (NES) | 1.5301898 |
| Nominal p-value | 0.026004728 |
| FDR q-value | 0.18191224 |
| FWER p-Value | 0.999 |
Table: GSEA Results Summary

  

Fig 1: Enrichment plot: GOMF\_OXIDOREDUCTASE\_ACTIVITY\_ACTING\_ON\_THE\_ALDEHYDE\_OR\_OXO\_GROUP\_OF\_DONORS      
 Profile of the Running ES Score & Positions of GeneSet Members on the Rank Ordered List

  

| SYMBOL | TITLE | RANK IN GENE LIST | RANK METRIC SCORE | RUNNING ES | CORE ENRICHMENT || 1 | PDHB | pyruvate dehydrogenase E1 subunit beta [Source:HGNC Symbol;Acc:HGNC:8808] | 149 | 0.862 | 0.0638 | Yes |
| 2 | DLD | dihydrolipoamide dehydrogenase [Source:HGNC Symbol;Acc:HGNC:2898] | 565 | 0.635 | 0.0871 | Yes |
| 3 | CYP27A1 | cytochrome P450 family 27 subfamily A member 1 [Source:HGNC Symbol;Acc:HGNC:2605] | 611 | 0.619 | 0.1378 | Yes |
| 4 | HSD17B12 | hydroxysteroid 17-beta dehydrogenase 12 [Source:HGNC Symbol;Acc:HGNC:18646] | 795 | 0.570 | 0.1734 | Yes |
| 5 | ALDH2 | aldehyde dehydrogenase 2 family member [Source:HGNC Symbol;Acc:HGNC:404] | 1003 | 0.517 | 0.2025 | Yes |
| 6 | ALDH3A2 | aldehyde dehydrogenase 3 family member A2 [Source:HGNC Symbol;Acc:HGNC:403] | 1263 | 0.465 | 0.2231 | Yes |
| 7 | ALDH18A1 | aldehyde dehydrogenase 18 family member A1 [Source:HGNC Symbol;Acc:HGNC:9722] | 1402 | 0.443 | 0.2511 | Yes |
| 8 | ALDH5A1 | aldehyde dehydrogenase 5 family member A1 [Source:HGNC Symbol;Acc:HGNC:408] | 1661 | 0.405 | 0.2664 | Yes |
| 9 | ALDH1A1 | aldehyde dehydrogenase 1 family member A1 [Source:HGNC Symbol;Acc:HGNC:402] | 1737 | 0.393 | 0.2950 | Yes |
| 10 | AKR1C3 | aldo-keto reductase family 1 member C3 [Source:HGNC Symbol;Acc:HGNC:386] | 1996 | 0.361 | 0.3066 | Yes |
| 11 | ALDH7A1 | aldehyde dehydrogenase 7 family member A1 [Source:HGNC Symbol;Acc:HGNC:877] | 2158 | 0.343 | 0.3241 | Yes |
| 12 | PDHA1 | pyruvate dehydrogenase E1 subunit alpha 1 [Source:HGNC Symbol;Acc:HGNC:8806] | 2433 | 0.310 | 0.3299 | Yes |
| 13 | BCKDHB | branched chain keto acid dehydrogenase E1 subunit beta [Source:HGNC Symbol;Acc:HGNC:987] | 2765 | 0.273 | 0.3280 | Yes |
| 14 | ALDH9A1 | aldehyde dehydrogenase 9 family member A1 [Source:HGNC Symbol;Acc:HGNC:412] | 2795 | 0.270 | 0.3494 | Yes |
| 15 | FAR2 | fatty acyl-CoA reductase 2 [Source:HGNC Symbol;Acc:HGNC:25531] | 2999 | 0.250 | 0.3555 | Yes |
| 16 | DHTKD1 | dehydrogenase E1 and transketolase domain containing 1 [Source:HGNC Symbol;Acc:HGNC:23537] | 3112 | 0.239 | 0.3677 | Yes |
| 17 | AKR1B1 | aldo-keto reductase family 1 member B [Source:HGNC Symbol;Acc:HGNC:381] | 3138 | 0.236 | 0.3864 | Yes |
| 18 | GAPDHS | "glyceraldehyde-3-phosphate dehydrogenase, spermatogenic [Source:HGNC Symbol;Acc:HGNC:24864]" | 3466 | 0.205 | 0.3790 | Yes |
| 19 | HAO1 | hydroxyacid oxidase 1 [Source:HGNC Symbol;Acc:HGNC:4809] | 3493 | 0.203 | 0.3947 | Yes |
| 20 | FMO5 | flavin containing dimethylaniline monoxygenase 5 [Source:HGNC Symbol;Acc:HGNC:3773] | 3759 | 0.181 | 0.3899 | No |
| 21 | ADH5 | "alcohol dehydrogenase 5 (class III), chi polypeptide [Source:HGNC Symbol;Acc:HGNC:253]" | 4720 | 0.094 | 0.3234 | No |
| 22 | ALDH6A1 | aldehyde dehydrogenase 6 family member A1 [Source:HGNC Symbol;Acc:HGNC:7179] | 5560 | 0.028 | 0.2606 | No |
| 23 | ALDH3B2 | aldehyde dehydrogenase 3 family member B2 [Source:HGNC Symbol;Acc:HGNC:411] | 5617 | 0.022 | 0.2582 | No |
| 24 | DLAT | dihydrolipoamide S-acetyltransferase [Source:HGNC Symbol;Acc:HGNC:2896] | 5647 | 0.020 | 0.2577 | No |
| 25 | BCKDHA | branched chain keto acid dehydrogenase E1 subunit alpha [Source:HGNC Symbol;Acc:HGNC:986] | 5671 | 0.018 | 0.2575 | No |
| 26 | ALDH1A3 | aldehyde dehydrogenase 1 family member A3 [Source:HGNC Symbol;Acc:HGNC:409] | 5831 | 0.007 | 0.2457 | No |
| 27 | AKR1C4 | aldo-keto reductase family 1 member C4 [Source:HGNC Symbol;Acc:HGNC:387] | 5843 | 0.005 | 0.2453 | No |
| 28 | ALDH3B1 | aldehyde dehydrogenase 3 family member B1 [Source:HGNC Symbol;Acc:HGNC:410] | 5862 | 0.004 | 0.2443 | No |
| 29 | AOX1 | aldehyde oxidase 1 [Source:HGNC Symbol;Acc:HGNC:553] | 6243 | -0.024 | 0.2168 | No |
| 30 | ALDH3A1 | aldehyde dehydrogenase 3 family member A1 [Source:HGNC Symbol;Acc:HGNC:405] | 6413 | -0.036 | 0.2068 | No |
| 31 | ALDH1B1 | aldehyde dehydrogenase 1 family member B1 [Source:HGNC Symbol;Acc:HGNC:407] | 6551 | -0.046 | 0.2002 | No |
| 32 | PDHX | pyruvate dehydrogenase complex component X [Source:HGNC Symbol;Acc:HGNC:21350] | 6760 | -0.060 | 0.1892 | No |
| 33 | ALDH8A1 | aldehyde dehydrogenase 8 family member A1 [Source:HGNC Symbol;Acc:HGNC:15471] | 7998 | -0.155 | 0.1066 | No |
| 34 | AKR1B10 | aldo-keto reductase family 1 member B10 [Source:HGNC Symbol;Acc:HGNC:382] | 8420 | -0.187 | 0.0902 | No |
| 35 | OGDH | oxoglutarate dehydrogenase [Source:HGNC Symbol;Acc:HGNC:8124] | 8503 | -0.194 | 0.1008 | No |
| 36 | ADH7 | "alcohol dehydrogenase 7 (class IV), mu or sigma polypeptide [Source:HGNC Symbol;Acc:HGNC:256]" | 8906 | -0.225 | 0.0892 | No |
| 37 | PDHA2 | pyruvate dehydrogenase E1 subunit alpha 2 [Source:HGNC Symbol;Acc:HGNC:8807] | 9124 | -0.241 | 0.0934 | No |
| 38 | ALDH1L1 | aldehyde dehydrogenase 1 family member L1 [Source:HGNC Symbol;Acc:HGNC:3978] | 9302 | -0.256 | 0.1021 | No |
| 39 | OGDHL | oxoglutarate dehydrogenase L [Source:HGNC Symbol;Acc:HGNC:25590] | 10754 | -0.399 | 0.0241 | No |
| 40 | GAPDH | glyceraldehyde-3-phosphate dehydrogenase [Source:HGNC Symbol;Acc:HGNC:4141] | 11268 | -0.467 | 0.0251 | No |
| 41 | ALDH1A2 | aldehyde dehydrogenase 1 family member A2 [Source:HGNC Symbol;Acc:HGNC:15472] | 11796 | -0.548 | 0.0321 | No |
| 42 | ALDH4A1 | aldehyde dehydrogenase 4 family member A1 [Source:HGNC Symbol;Acc:HGNC:406] | 12080 | -0.614 | 0.0638 | No |
Table: GSEA details [plain text format]

  

Fig 2: GOMF\_OXIDOREDUCTASE\_ACTIVITY\_ACTING\_ON\_THE\_ALDEHYDE\_OR\_OXO\_GROUP\_OF\_DONORS      
 Blue-Pink O' Gram in the Space of the Analyzed GeneSet

  

Fig 3: GOMF\_OXIDOREDUCTASE\_ACTIVITY\_ACTING\_ON\_THE\_ALDEHYDE\_OR\_OXO\_GROUP\_OF\_DONORS: Random ES distribution      
 Gene set null distribution of ES for **GOMF\_OXIDOREDUCTASE\_ACTIVITY\_ACTING\_ON\_THE\_ALDEHYDE\_OR\_OXO\_GROUP\_OF\_DONORS**
